# Roles of Calcium Signaling in Gene Expression and Photosynthetic Acclimatization of *Solanum lycopersicum* Micro-Tom (MT) after Mechanical Damage

**DOI:** 10.3390/ijms232113571

**Published:** 2022-11-05

**Authors:** Felipe Girotto Campos, Diana Pacheco Seixas, Gustavo Ribeiro Barzotto, Letícia Galhardo Jorge, Karina Renostro Ducatti, Gisela Ferreira, Tatiane Maria Rodrigues, Edvaldo Aparecido Amaral da Silva, Carmen Sílvia Fernandes Boaro

**Affiliations:** 1 Biostatistics, Plant Biology, Parasitology and Zoology Department, Institute of Biosciences, São Paulo State University (UNESP), Campus Botucatu, Street Prof. Dr. Antônio Celso Wagner Zanin 250, Botucatu 18618-970, SP, Brazil; 2 Plant Production Department, School of Agriculture, São Paulo State University (UNESP), Campus Botucatu, Av. Universitária n° 3780, Altos do Paraíso, Botucatu 18610-307, SP, Brazil

**Keywords:** chlorophyll *a* fluorescence, gas exchange, hydrogen peroxide, plant stress, *RBOH1* genes

## Abstract

A momentary increase in cytoplasmic Ca^2+^ generates an oscillation responsible for the activation of proteins, such as calmodulin and kinases, which interact with reactive oxygen species (ROS) for the transmission of a stress signal. This study investigated the influence of variations in calcium concentrations on plant defense signaling and photosynthetic acclimatization after mechanical damage. *Solanum lycopersicum* Micro-Tom was grown with 0, 2 and 4 mM Ca^2+^, with and without mechanical damage. The expression of stress genes was evaluated, along with levels of antioxidant enzymes, hydrogen peroxide, lipid peroxidation, histochemistry, photosynthesis and dry mass of organs. The ROS production generated by mechanical damage was further enhanced by calcium-free conditions due to the inactivation of the oxygen evolution complex, contributing to an increase in reactive species. The results indicated that ROS affected mechanical damage signaling because calcium-free plants exhibited high levels of H_2_O_2_ and enhanced expression of kinase and *RBOH1* genes, necessary conditions for an efficient response to stress. We conclude that the plants without calcium supply recognized mechanical damage but did not survive. The highest expression of the *RBOH1* gene and the accumulation of H_2_O_2_ in these plants signaled cell death. Plants grown in the presence of calcium showed higher expression of *SlCaM2* and control of H_2_O_2_ concentration, thus overcoming the stress caused by mechanical damage, with photosynthetic acclimatization and without damage to dry mass production.

## 1. Introduction

Mechanical damage in plants activates a cascade of defense reactions closely related to plant defenses against herbivory [1]. Mechanically damaged plant tissues produce O_2_ and H_2_O_2_, which are ROS involved in a wide range of biological processes such as growth, development and responses to biotic and abiotic stimuli [2,3,4]. Increased ROS production promotes Ca^2+^-channel opening, resulting in increased cytoplasmic Ca^2+^ [5,6]. This cytoplasmic calcium activates the respiratory burst oxidase homolog-D (RBOHD), the enzyme responsible for ROS wave formation, thus aiding ROS propagation from stimulated to unstimulated tissues during stress [6,7].

A momentary increase in cytoplasmic Ca^2+^ generates an oscillation responsible for the activation of proteins such as calmodulin (CaMs), CaM-likes, calcinerin B-like and calcium-dependent protein kinases (CDPKs) [8]. High levels of Ca^2+^ and ROS activate MAPKs (mitogen-activated protein kinases) and calmodulin to regulate ROS accumulation [5]. Furthermore, expression of the calmodulin gene (*SlCaM2*) present in tomato leaves is dependent on, among other factors, mechanical damage, an important intermediate in signaling abiotic and biotic stresses, along with kinases [9].

Calcium-dependent calmodulins play an important role in signaling and activating kinases in response to stress [10] since MAPKs translate extracellular stimuli to intracellular responses [1]. Calcium application activates Calmodulin, provides better coordination and stimulates antioxidant enzymes, which reduce lipid peroxidation, allowing tolerance to stress induced by, for example, the element nickel [10,11,12]. The signals of calcium trigger transduction for gene expression and activation of biochemical responses, which enable faster and more efficient neutralization of ROS by antioxidant enzymes, thereby protecting photosystems and maintaining a high photosynthetic rate [13].

Among kinases activated by calmodulins, MAPKs are signaling modules conserved in eukaryotes that link the perception of stimuli to cellular responses. This signaling combines at least three sequentially phosphorylation-activated protein kinases, *MAP3K* and *MAP2K* [14]. In plants, MAPK cascades are mainly associated with drought, salinity, cold, injury, ozone, ROS and hormonal stimuli, as well as developmental processes such as cell division and differentiation or abscission [15,16]. In *Arabidopsis*, *MPK1* and *MPK2* are activated by stresses induced by wounding and H_2_O_2_ [17].

Specifically, it is known that stress conditions caused by cold, drought and Paraquat^®®^ application induce increased *MPK1* and *MPK2* transcription in leaves of tomato plants inoculated with tobacco mosaic virus. In addition, silencing of the *RBOH1* gene reduced the expression of *MPK1* and *MPK2* genes by 70%, suggesting that H_2_O_2_ from *RBOH1* in the apoplast is essential for the activation of *MPK1* and *MPK2* [18].

RBOHD regulation by C-terminal ubiquinone phosphorylation is mediated by calcium-dependent protein kinases (CPKs), also preserved in plants and animals due to H_2_O_2_ synthesis regulation, and is related to cell death [19,20,21,22]. *Arabidopsis* mutants kept in the dark and with RBOHD absence or overexpression revealed that RBOHD is responsible for damage recognition and H_2_O_2_ control [23].

The mentioned genes are directly related to ROS, calcium signaling pathways and stress responses. In this way, the evaluation of gene expression can provide evidence of mechanical damage interfering with the use of endogenous calcium to overcome stress at the expense of photosynthesis, which also requires calcium [24].

Stress by calcium deficiency has been found to interfere with the photosynthetic process, such as with electron flow, since it destabilizes the oxygen evolution complex and causes greater energy dissipation in the form of heat by the antennas (*D*), as observed in corn and tomatoes plant [24].

On the other hand, exogenous calcium in *Pennisetum americanum* (L.) K. Schum was found to contribute to the regulation of low H_2_O_2_ levels, allowing the restoration of chlorophyllous tissues, maintaining high expression of the D1 protein, and adjusting glutamine-S-transferase, which prevented photoinhibition of PSII [25].

There are reports of the involvement of calcium–calmodulin in the synthesis of violaxanthine deepoxidase, which contributes to the xanthophyll cycle, an important pathway for the dissipation of non-photochemical energy (NPQ). Thus, plants with calcium deficiency and under conditions of abiotic stress can direct this element for signaling and stress response pathways, which can lead to energy accumulation in photosystem II, thereby limiting the photosynthetic process [26].

*Solanum lycopersicum* Micro-Tom (MT) is a model plant for studies of gene expression and physiological processes [27] since its genome is sequenced and its signaling pathways are well known. This condition allows the investigation of the roles of Ca^2+^ and H_2_O_2_ in signaling and gene expression involved with antioxidant enzyme activity, thereby contributing to the maintenance of the photosynthetic process in plants subjected to mechanical damage.

Therefore, this study aimed to investigate the influence of variation in calcium concentration on signaling of defense against mechanical damage, as well as restoration and photosynthetic acclimation, using *Solanum lycopersicum* Micro-Tom (MT) as a model plant.

## 2. Results

### 2.1. Differentiated Expression of RBOH1, MPK1, MPK2 and SlCaM2 under Ca^2+^ Presence/Absence (Ca^2+^-Free) and with/without Mechanical Damage

**Figure 1:**
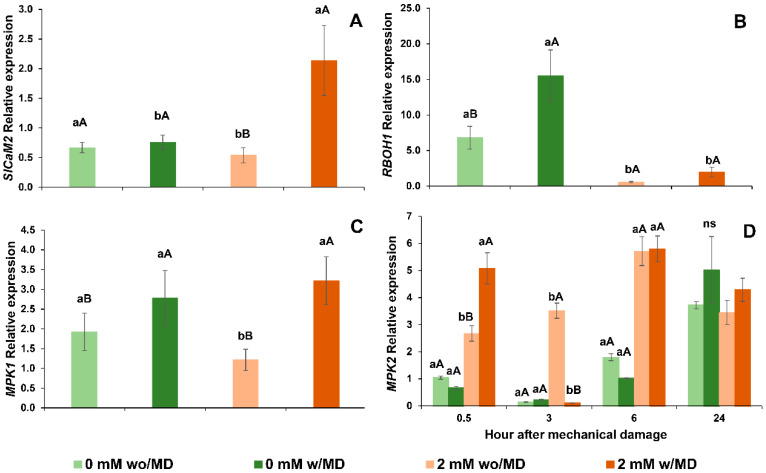
Gene expression, (**A**). *SlCaM2* damage x calcium *p* < 0.005, (**B**). *RBOH1* calcium × damage *p* < 0.016, (**C**). *MPK1* damage × calcium *p* < 0.023 and (**D**). *MPK2* time × damage × calcium *p* < 0.027 in *Solanum lycopersicum* “cv. Micro-Tom” grown with variation in calcium concentration (mM) and with (w/MD) or without (wo/MD) mechanical damage. Different lowercase letters indicate a significant difference in calcium levels within wo/MD or w/MD plants. Capital letters test wo/MD and w/MD plants within the same calcium level. ns = not significant at 5% Tukey test. Bars correspond to averages, whiskers to ± SE (*n* = 3).

The time since mechanical damage was not significant (*p* < 0.05), except for *MPK2*, and so only calcium and mechanical damage showed an interaction, and are thus represented in Figure 1.

Calcium and mechanical damage contributed to increased *SlCaM2* expression (Figure 1A), whereas the absence of Ca^2+^ with mechanical damage increased *RBOH1* expression (Figure 1B). *MPK1* showed higher gene expression in plants with mechanical damage, independent of Ca^2+^ level (Figure 1C). However, *MPK2* showed higher gene expression in plants with Ca^2+^ at 0.5 h and with mechanical damage, indicating that the presence of calcium increased *MPK2* expression (Figure 1D).

### 2.2. Ca^2+^ Absence with Mechanical Damage Activated Antioxidant Enzymes and Increased Hydrogen Peroxide and Lipid Peroxidation

**Figure 2:**
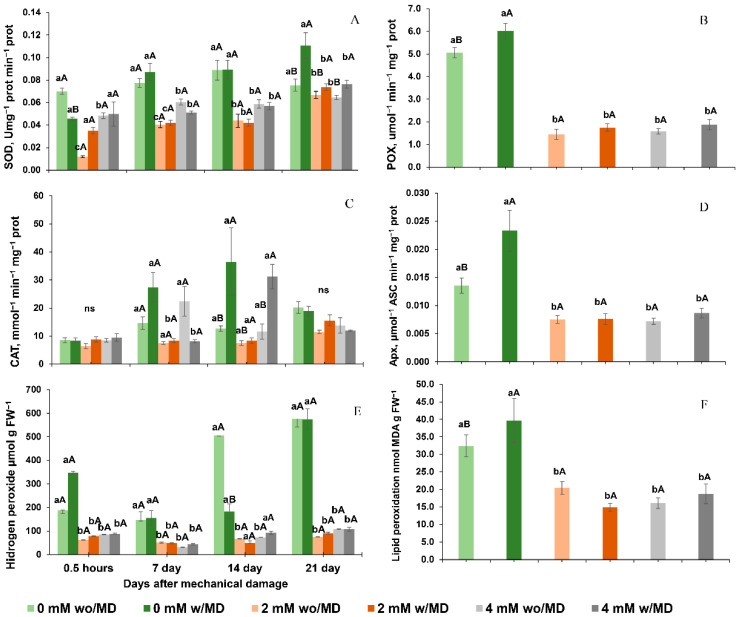
Activity of antioxidants (**A**). Superoxide dismutase (SOD) time × damage × calcium *p* < 0.001, (**B**). Peroxidase (POX) damage × calcium *p* < 0.045 and (**C**). Catalase (CAT) time × damage × calcium *p* < 0.033 and (**D**). Ascorbate peroxidase (APX) damage × calcium *p* < 0.006, (**E**). Hydrogen peroxide (H_2_O_2_) concentration time × damage × calcium *p* < 0.001 and (**F**). Lipid peroxidation (expressed by the formation of malonaldehyde, MDA) damage × calcium *p* < 0.017, in *Solanum lycopersicum* “cv. Micro-Tom” grown with variation in calcium concentration (mM) and with (w/MD) or without (wo/MD) mechanical damage. Different lowercase letters indicate a significant difference in calcium levels within wo/MD or w/MD plants. Capital letters test wo/MD and w/MD plants within the same calcium level. ns = not significant at 5% Tukey test. Bars correspond to averages, whiskers to ± SE (*n* = 3).

The time since mechanical damage was not significant (*p* < 0.05), except for SOD, CAT and hydrogen peroxide, and so only calcium and mechanical damage showed an interaction, and are thus represented in Figure 2.

In general, Ca^2+^-free plants had higher activities of the enzymes SOD, POD and APX when subjected to mechanical damage. In general, there was no variation in the activities of these enzymes with 2 and 4 mM Ca^2+^, independent of mechanical damage (Figure 2A,B,D). Specifically, the highest activities of the enzyme CAT were for Ca^2+^-free and 4 mM Ca^2+^-cultivated plants, with and without damage (Figure 2C).

Plants grown with 2 and 4 mM Ca^2+^ showed no variation in hydrogen peroxide concentration, regardless of mechanical damage, and had the lowest concentrations. Plants grown in the absence of Ca^2+^ and with mechanical damage presented a higher concentration of hydrogen peroxide immediately after damage, and its concentration varied little in subsequent evaluations (Figure 2E).

Lipid peroxidation was higher for Ca^2+^-free plants with mechanical damage. Lipid peroxidation did not differ among plants grown in the presence of Ca^2+^ (Figure 2F).

Plants grown in the absence of Ca^2+^ and with mechanical damage resisted the deficiency of the element longer.

### 2.3. Hydrogen Peroxide Was Evidenced in Leaves of Plants Grown in the Presence of Ca^2+^ and with Mechanical Damage

**Figure 3:**
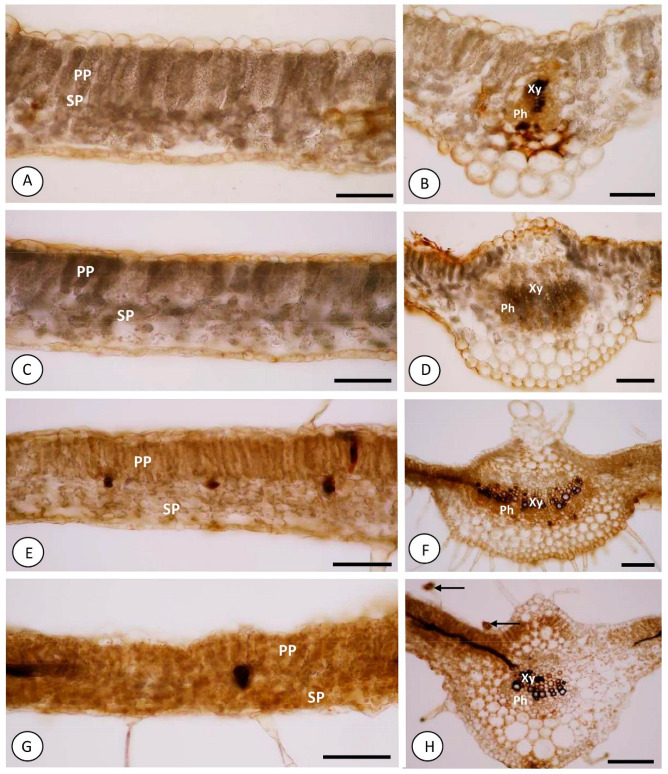
Photomicrographs of the median cross sections of the leaf blade of *Solanum lycopersicum* “cv. Micro-Tom” stained with 3,3′-diaminobenzidine (DAB) at 0.5 h after mechanical damage. (**A**,**B**). absence of Ca^2+^ and without mechanical damage. (**C**,**D**). absence of Ca^2+^ and with mechanical damage. (**E**,**F**). presence of 2 mM Ca^2+^ and without mechanical damage. (**G**,**H**). presence of 2 mM Ca^2+^ and with mechanical damage. (**A**,**C**,**E**,**G**). Mesophyll region. (**B**,**D**,**F**,**H**). Midrib. PP—Palisade parenchyma; SP—Spongy parenchyma; Xy—Xylem; Ph—Phloem; ↑ (arrow)—Glandular trichome. Bars: ((**A**–**C**,**E**,**G**) = 100 μm); ((**D**,**F**,**H**) = 150 μm).

Thirty minutes after mechanical damage, Ca^2+^-free plants without mechanical damage showed discrete staining from reactive 3,3′-diaminobenzidine (DAB) restricted to the walls of parenchymal cells of the endoderm on the abaxial surface of the midrib, as well as the phloem and vessel elements of the midrib and smaller veins (Figure 3A–D). No staining was observed in the mesophyll cells under these conditions.

Plants grown in the presence of 2 mM Ca^2+^ and without mechanical damage had staining within some cells of the palisade parenchyma and spongy parenchyma (Figure 3E). There was intense DAB staining on the walls of the vessel elements of the midrib (Figure 3F) and of the smaller veins. Otherwise, plants grown in the presence of 2 mM Ca^2+^ and with mechanical damage revealed more intense hydrogen peroxide staining by DAB inside some palisade and spongy parenchyma cells (Figure 3G) and in the head cells of glandular trichomes (Figure 3H). Staining on the walls of vessel elements of the midrib (Figure 3H) and in the smaller veins was also observed.

**Figure 4:**
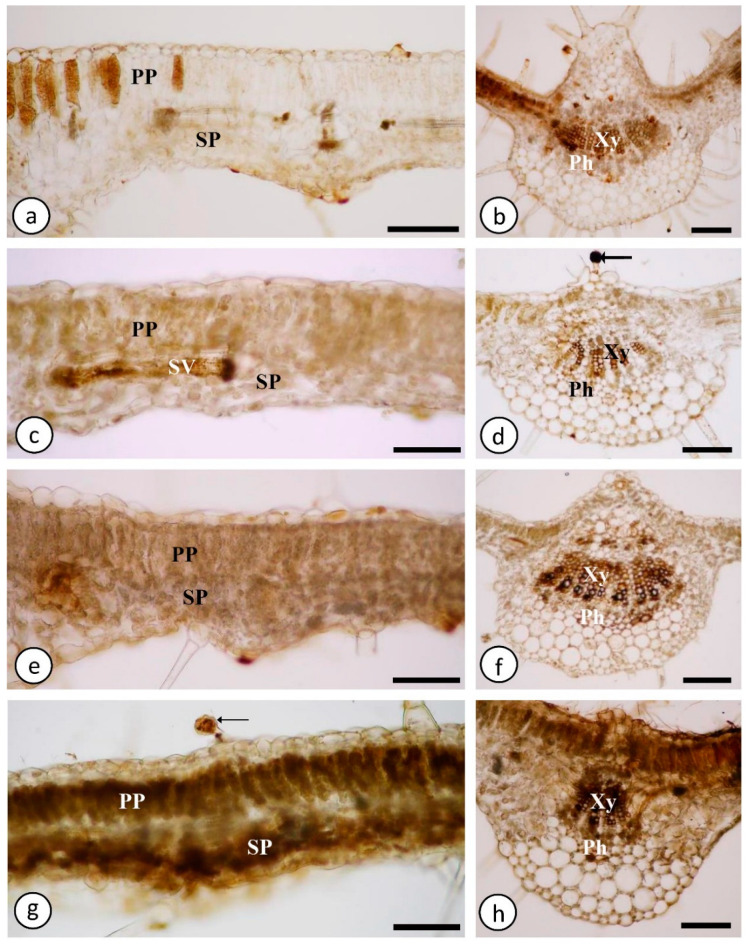
Photomicrographs of the median cross sections of the leaf blade of *Solanum lycopersicum* “cv Micro-Tom” stained with 3,3′-diaminobenzidine (DAB) at 21 days after mechanical damage. (**a**,**b**). absence of Ca^2+^ and without mechanical damage. (**c**,**d**). absence of Ca^2+^ and with mechanical damage. (**e**,**f**). presence of 2 mM Ca^2+^ and without mechanical damage. (**g**,**h**). presence of 2 mM Ca^2+^ with mechanical damage. (**a**,**c**,**e**,**g**). Mesophyll region. (**b**,**d**,**f**,**h**). Midrib. PP—Palisade parenchyma; SP—Spongy parenchyma; Xy—Xylem; Ph—Phloem; SV—Smaller veins; ↑ (arrow)—Glandular trichome. Bars: ((**c**,**e**,**g**) = 100 μm); ((**a**,**b**,**d**,**f**,**h**) = 150 μm).

At 21 days, Ca^2+^-free plants without mechanical damage showed subtle staining for hydrogen peroxide within some cells of the palisade parenchyma (Figure 4a) and on the walls of vessel elements of the midrib (Figure 4b) and of smaller veins, while Ca^2+^-free plants with mechanical damage showed staining on the walls of the vessel elements of the smallest veins (Figure 4c) and the midrib (Figure 4d). Staining was also detected inside the head cells of glandular trichomes (Figure 4d).

Plants grown in the presence of 2 mM Ca^2+^ and without mechanical damage showed intense staining for hydrogen peroxide on the walls of the vessel elements of the midrib (Figure 4f) and smaller veins. No staining of parenchymal cells was observed in the mesophyll (Figure 4e). Plants grown in the presence of 2 mM Ca^2+^ and with mechanical damage had more intense and generalized staining for hydrogen peroxide. The staining was evident in the head cells of glandular trichomes (Figure 4g), inside palisade and spongy parenchyma cells (Figure 4g) and on the walls of the vessel elements of the midrib (Figure 4h) and smaller veins.

Cross sections of the mesophyll region and midrib of a leaf blade of *Solanum lycopersicum* “cv. Micro-Tom” without DAB treatment are shown in Supplementary Figure S1.

### 2.4. Performance of Chlorophyll a Fluorescence Does Not Differ between Plants Grown in the Absence of Ca^2+^ with and without Damage, but There Is an Influence Resulting in Low Gas Exchange and Plant Biomass

**Figure 5:**
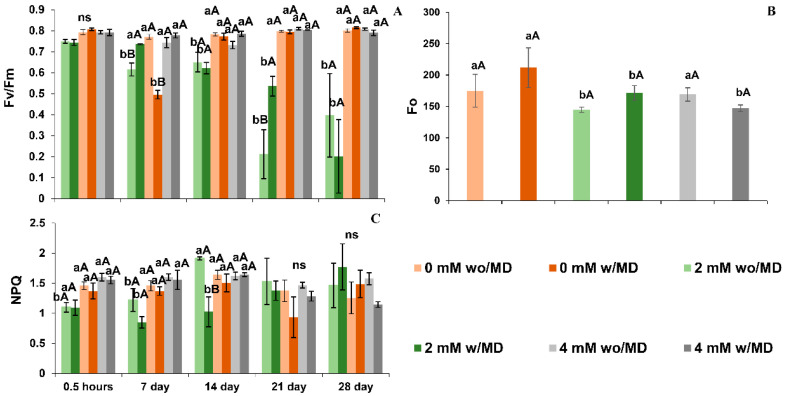
(**A**). Maximum quantum efficiency of photosystem II (Fv/Fm) time × damage × calcium *p* < 0.007; (**B**). minimum dark-adapted fluorescence (Fo) damage × calcium *p* < 0.028; (**C**). non-photochemical quenching (NPQ) time × damage × calcium *p* < 0.033 in *Solanum lycopersicum* “cv. Micro-Tom” grown with variation in calcium concentration (mM) and with (w/MD) or without (wo/MD) mechanical damage. Different lowercase letters indicate a significant difference in calcium levels within wo/MD or w/MD plants. Capital letters test wo/MD and w/MD plants within the same calcium level. ns = not significant at 5% Tukey test. Bars correspond to averages, whiskers to ± SE (*n* = 3).

**Figure 6:**
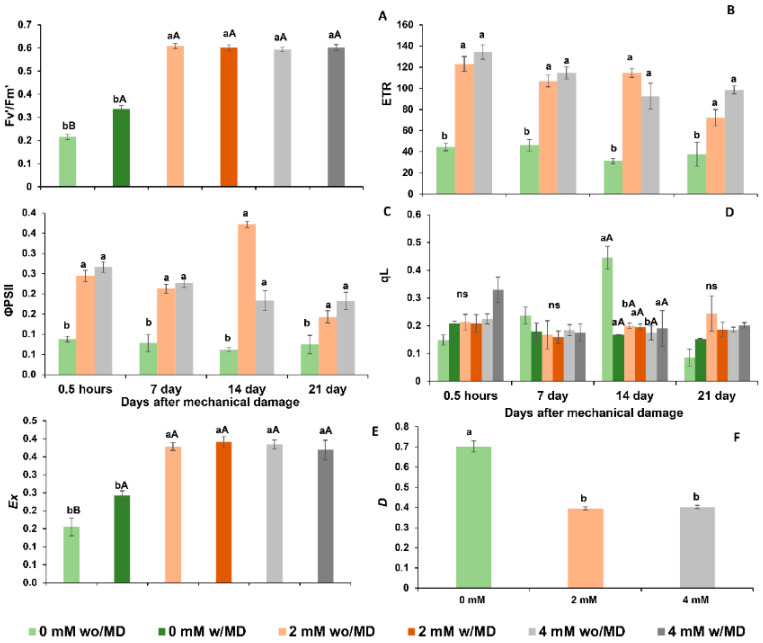
(**A**). Photosystem II efficiency (Fv’/Fm’) damage × calcium <0.001; (**B**). electron transport rate (ETR) time × calcium *p* < 0.008; (**C**). effective quantum yield (ΦPSII) time × calcium *p* < 0.008; (**D**). photochemical quenching (qL) time × damage × calcium *p* < 0.002; (**E**). fraction of dissipated excitation energy in the antenna that cannot be used for photochemical phase (*Ex*) damage × calcium *p* < 0.005; (**F**). light fraction absorbed by the PSII antenna that is dissipated as heat (*D*) calcium *p* < 0.001, in *Solanum lycopersicum* “cv. Micro-Tom” grown with variation in calcium concentration (mM) and with (w/MD) or without (wo/MD) mechanical damage. Different lowercase letters indicate a significant difference in calcium levels within wo/MD or w/MD plants. Capital letters test wo/MD and w/MD plants within the same calcium level. ns = not significant at 5% Tukey test. Bars correspond to averages, whiskers to ± SE (*n* = 3).

Plants grown in the absence of Ca^2+^ with and without mechanical damage did not differ in chlorophyll *a* fluorescence performance (Figure 5 and Figure 6). Likewise, plants grown in the presence of 2 or 4 mM Ca^2+^ and with and without damage did not differ in chlorophyll *a* fluorescence performance (Figure 5 and Figure 6).

Plants of *Solanum lycopersicum* cv. Micro-Tom grown in the absence of Ca^2+^ and with or without mechanical damage, in the dark, showed decreased effective quantum efficiency (ΦPSII), electron transport rate (ETR), potential quantum efficiency (Fv/Fm) and PSII efficiency (Fv’/Fm’) over time, with a high fraction of light absorbed by the PSII antenna and dissipated as heat (*D*) (Figure 5 and Figure 6). In the presence of light, these same plants showed low PSII efficiency (Fv’/Fm’), photochemical quenching (qL), electron transport rate (ETR), and effective quantum efficiency (ΦPSII), and the fraction of excitation energy did not dissipate in the antenna. It did not use photochemistry (*Ex*), and with the fraction of light absorbed by the PSII antenna, it dissipated most as heat (*D*) (Figure 5 and Figure 6). Plants grown in the presence of 2 and 4 mM of Ca^2+^ and with or without mechanical damage presented high non-photochemical extinction coefficients (NPQ) throughout the evaluations (Figure 5 and Figure 6).

**Figure 7:**
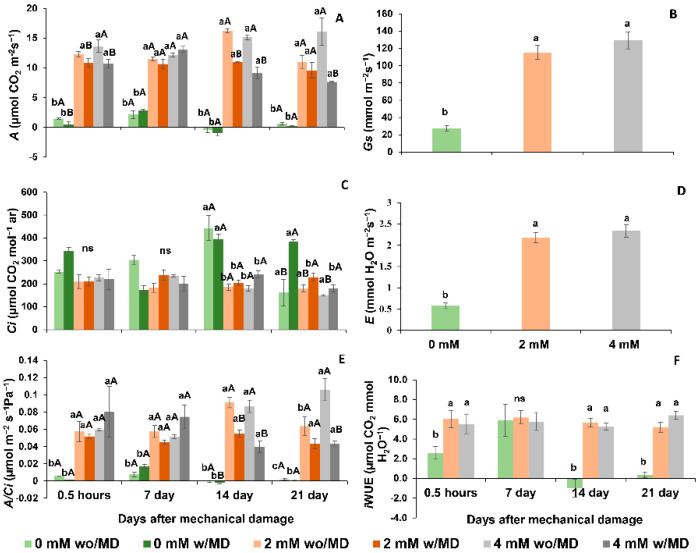
(**A**). CO_2_ assimilation rate (*A*) time × damage × calcium *p* < 0.005; (**B**). stomatal conductance (*Gs*) calcium *p* < 0.001; (**C**). internal leaf CO_2_ concentration (*Ci*) time × damage × calcium *p*< 0.001; (**D**). transpiration rate (*E*) calcium *p* < 0.001; (**E**). Instant carboxylation efficiency of ribulose enzyme 1,5-diphosphate carboxylase (RuBisCO) (*A*/*Ci*) time × damage × calcium *p* < 0.016; (**F**). water use efficiency (iWUE) time × calcium *p* < 0.001 in *Solanum lycopersicum* “cv. Micro-Tom” grown with variation in calcium concentration (mM) and with (w/MD) or without (wo/MD) mechanical damage. Different lowercase letters indicate a significant difference in calcium levels within wo/MD or w/MD plants. Capital letters test wo/MD and w/MD plants within the same calcium level. ns = not significant at 5% Tukey test. Bars correspond to averages, whiskers to ± SE (*n* = 3).

**Figure 8:**
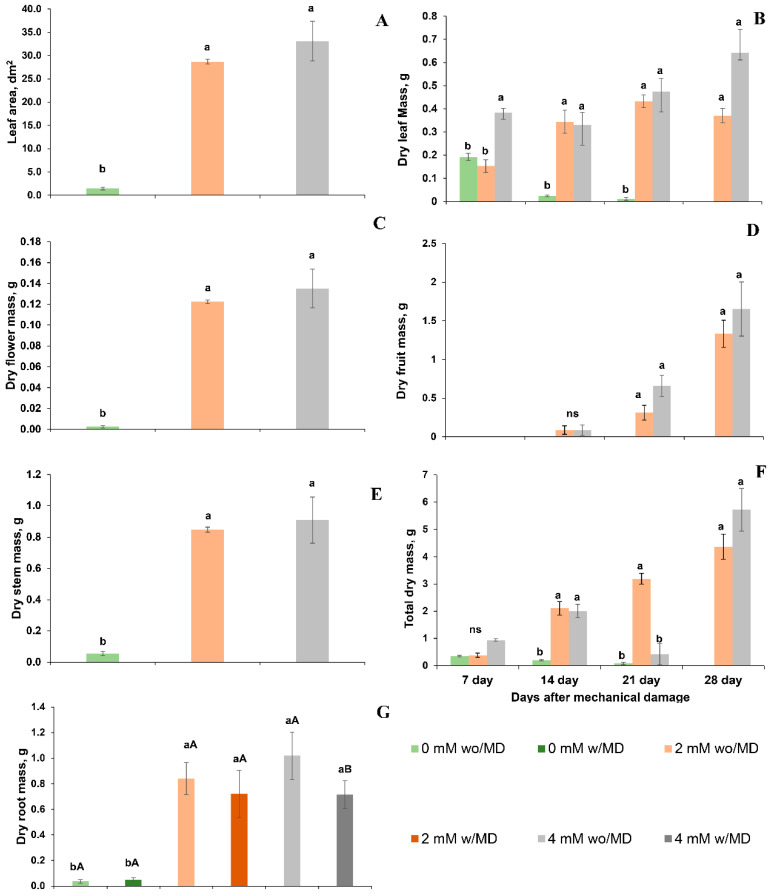
(**A**). Leaf area (dm^2^) damage × calcium *p* < 0.011; (**B**). leaf dry mass (g) time × damage × calcium *p* < 0.004; (**C**). flower dry mass (g) damage × calcium *p* < 0.022; (**D**). fruit dry mass (g) time × calcium *p* < 0.037; (**E**). stem dry mass (g) damage × calcium *p* < 0.008; (**F**). root dry mass (g) damage × calcium *p* < 0.010; (**G**). total dry mass (g) time × damage × calcium *p* < 0.001, in *Solanum lycopersicum* “cv. Micro-Tom” grown with variation in calcium concentration (mM) and with (w/MD) or without (wo/MD) mechanical damage. Different lowercase letters indicate a significant difference in calcium levels within wo/MD or w/MD plants. Capital letters test wo/MD and w/MD plants within the same calcium level. ns = not significant at 5% Tukey test. Bars correspond to averages, whiskers to ± SE (*n* = 3).

Plants grown in the absence of Ca^2+^ and with or without mechanical damage showed low carbon assimilation (*A*), stomatal conductance (*Gs*), transpiration (*E*), instantaneous water use efficiency (iWUE), the efficiency of RuBisCO carboxylation (*A*/*Ci*) and high concentrations of intracellular carbon (*Ci*) (Figure 7). These conditions probably contributed to smaller leaf area and lower total dry mass and lower root, stem, leaf, flower and fruit dry mass (Figure 8). It is noteworthy that Ca^2+^-free plants without mechanical damage died before Ca^2+^-free plants with mechanical damage.

Plants grown in the presence of 2 and 4 mM of Ca^2+^ and without damage had similar carbon assimilation rates, RuBisCO carboxylation and water use efficiencies. Moreover, as revealed at the end of the evaluations, these plants had similar total dry mass and fruit and stem dry mass (Figure 8), although leaf and root dry mass were higher for plants grown in the presence of 4 mM Ca^2+^.

Plants grown in the presence of 2 and 4 mM of Ca^2+^ and with mechanical damage showed lower carbon assimilation rates, RuBisCO carboxylation and water use efficiencies, as well as low stem, fruit and total dry mass (Figure 7 and Figure 8), compared to the same without mechanical damage.

Thus, the results for chlorophyll *a* fluorescence, gas exchange and plant biomass reveal that the photosynthetic performance of Ca^2+^-free plants with mechanical damage differs from that of those grown in the presence of Ca^2+^ and with mechanical damage. The *Ex* of Ca^2+^-free plants with mechanical damage was low, while that of plants grown in the presence of Ca^2+^ with mechanical damage was elevated (Figure 6). The *A*/*Ci* of Ca^2+^-free plants with mechanical damage was higher than that of Ca^2+^-free plants without mechanical damage seven days after damage (Figure 7). The *A*/*Ci* of plants grown in the presence of 2 and 4 mM Ca^2+^ and with mechanical damage was lower than that of plants grown in the presence of 2 and 4 mM Ca^2+^ and without mechanical damage at 14, 21 and 28 days after mechanical damage.

### 2.5. Two Clusters Were Found, One for Plants Grown in the Absence of Ca^2+^ and One for Those Grown in the Presence of Ca^2+^, and a Positive Correlation between H_2_O_2_, Gene Expression and Enzyme Activity Is Highlighted

**Figure 9:**
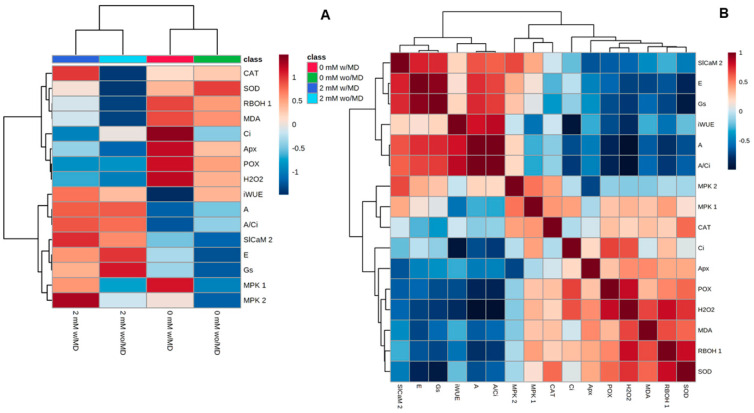
(**A**). Heat map and (**B**). Correlation analysis with expression of *RBOH1*, *MPK1*, *MPK2*, *SlCaM2* genes; enzymatic activity of SOD, POD, CAT and APX; hydrogen peroxide concentration; malonaldehyde; carbon assimilation; stomatal conductance; transpiration; internal carbon concentration; water use efficiency; and carboxylation efficiency of RuBisCO, in *Solanum lycopersicum* “cv. Micro-Tom” grown with variation in calcium concentration (mM) and with (w/MD) or without (wo/MD) mechanical damage at 0.5 h after damage. Values correspond to average ± SE (*n* = 3).

Hierarchical cluster analysis (HCA) revealed the formation of two clusters representing treatments with the absence of Ca^2+^ and with the presence of Ca^2+^. The presence of Ca^2+^ with mechanical damage exhibited a strong positive correlation with *SlCaM2* and *MPK1* expression and CAT enzyme activity. On the other hand, the absence of Ca^2+^ with mechanical damage exhibited a strong positive correlation with H_2_O_2_ and *RBOH1* and *MPK1* expression, POX and APX activity, lipid peroxidation and *Ci*, and a strong negative correlation with iWUE and *A*/*Ci* (Figure 9A).

The paired correlation heat map revealed that H_2_O_2_ is positively correlated with *RBOH1* and *MPK1* expression, SOD and POX activity, lipid peroxidation (MDA) and *Ci*, and negatively correlated with *SlCaM2* and *MPK2* expression and *E*, *Gs*, *A*, *A*/*Ci* and iWUE (Figure 9B).

## 3. Discussion

Plants of *Solanum lycopersicum* “cv. Micro-Tom” grown in the absence of Ca^2+^ showed signs of element deficiency, according to those recorded by Kalaji et al. [24] and Tang and Luan [28]. Several studies have revealed that plants grown in the absence of Ca^2+^ and with mechanical damage show greater expression of the *RBOH1* gene after mechanical damage. This gene is activated by calcium, and the protein it expresses is responsible for the production and control of hydrogen peroxide, which is involved in signaling and signal propagation of plant defenses [29]. These studies show that *RBOH1* is involved with injury recognition and the rapid and systemic cell-to-cell signaling induced by injury [20,21,22,23].

Accompanied by, and dependent on, the production and accumulation of H_2_O_2_ in extracellular spaces [4], the signaling described above can be converted into a radial signal, propagated among xylem cells by the release of Ca^2+^ from transporter glutamate receptor-like channels (GRLs), which is a mechanism that can interconnect signals generated by Ca^2+^ and reactive oxygen species (ROS) [30]. Accordingly, H_2_O_2_ markings by DAB suggest that this signal is propagated via xylem in plants grown in the absence of Ca^2+^ and with mechanical damage, a condition that suggests that *RBOH1* signal propagation may depend on, and be linked to, the presence of hydrogen peroxide in the absence of calcium. It is important to note that chloroplasts are a source of Ca^2+^ [31], and it was necessary for plants to use the element for the activation of *RBOH1*.

The heat map showed a positive influence on the expression of *RBOH1*, *MPK1* and *MPK2* in plants grown in the presence of 2 mM Ca^2+^ and with mechanical damage, compared to plants grown in the presence of 2 mM Ca^2+^ and without mechanical damage, which revealed a low concentration of H_2_O_2_, efficiently controlled by CAT. Plants grown in the absence of Ca^2+^- and with mechanical damage showed high expression of *RBOH1* and accumulation of H_2_O_2_. However, the increase in activity of the chloroplast enzyme APX should be noted, as it indicates an alteration possibly due to the activation of *RBOH1* with the use of Ca^2+^. The change in nutrient utilization priority negatively influenced photosynthesis with energy accumulation in the photosystem, which may have caused an increase in APX activity.

Plants grown in the absence of Ca^2+^ and without damage showed a negative influence on gas exchange, despite the low *Ci*. It is important to highlight the low correlation between *Gs* and *Ci*, which suggests stress in the photosynthetic system, probably due to the need for Ca^2+^ in the chloroplast for signaling and increasing APX and POX at the expense of SOD and CAT. Plants grown in the absence of Ca^2+^ and with mechanical damage showed higher *Ci*, which suggests the need for Ca^2+^ in the electron transport chain for CO_2_ incorporation.

Plants grown in the presence of 2 mM Ca^2+^ and with mechanical damage showed higher CAT activity, which controlled H_2_O_2_.

In plants grown in the absence of Ca^2+^ and with mechanical damage, a higher concentration of H_2_O_2_ may have contributed to the signaling of defense mechanisms, with the expression of *MPK1*, which, by acting in cascade, activates *MPK2*. This is supported by the positive correlations between H_2_O_2_ and *MPK1* and between *MPK1* and *MPK2*, which are genes involved in the recognition and overcoming of stress [15,16]. These genes transcribe *MPK1* and *MPK2* kinases involved with the activation of antioxidant enzymes, which may explain the greater activity of antioxidant enzymes in plants grown in the absence of calcium and with mechanical damage. In addition, it is known that silencing of *MPK1* and *MPK2* can result in decreased levels of Cu/Zn-SOD, APX, GR1 and CAT1 transcription [18].

In addition, Ca^2+^-free plants with mechanical damage had no activation of calmodulin, as the heat map reveals lower expression of the *SICaM2* gene, probably influenced by the damage. Calmodulin is necessary for the activation of *MPK2* and catalase, the first enzyme to neutralize H_2_O_2_, which revealed low activity in these plants right after mechanical damage. Even though catalase increased during the evaluations, it did not control the H_2_O_2_ concentration in these plants, as confirmed by the presence of hydrogen peroxide in plant tissue as indicated by DAB, which may have been responsible for the accumulation of malondialdehyde because of lipid peroxidation. Studies have demonstrated the need for calmodulin activation to coordinate the response to mechanical damage by activating MAPK kinases and antioxidant enzymes involved in regulating H_2_O_2_ levels [5,10,32,33].

Explained by the greater activity of peroxidases in plants with mechanical damage, the marking of hydrogen peroxide by DAB in Ca^2+^-free plants with mechanical damage was acuter than in Ca^2+^-free plants without mechanical damage. The DAB markings still suggest the need for the Ca^2+^ ion in the activation of peroxidases that neutralize H_2_O_2_ in the cell walls of the main veins. Studies have reported that calcium application stimulates cell wall peroxidase activity [34,35,36].

Plants grown in the absence of Ca^2+^ and without mechanical damage did not present stress response mechanisms, and the expression of target genes was not detected. The H_2_O_2_ concentration in these plants increased with time but was lower than in Ca^2+^-free plants with damage and was insufficient to signal stress and activate the genes (*RBOH1*, *SlCaM2*, *MPK1* and *MPK2*) necessary for CAT activation for H_2_O_2_ neutralization, resulting in structural damage such as malondialdehyde accumulation and tissue necrosis.

The absence of Ca^2+^ also interfered with the photosynthetic process and the accumulation of dry mass. *Solanum lycopersicum* plants grown in the absence of Ca^2+^, regardless of mechanical damage, showed impairment in the functioning of photosystems with decreases in potential quantum yield (Fv/Fm), photosystem II efficiency in light (Fv’/Fm’) and photochemical quenching in light (qL). These results indicate difficulty in capturing light and suggest that calcium deficiency interferes with the stability of photosystems [37,38].

In addition, the low effective quantum yield (ΦPSII) and electron transport rate (ETR) in Ca^2+^-free plants suggest that calcium deficiency in electron transport is influencing quantum productivity responsible for reducing carbon due to its channel energy. It has been suggested that calcium is important for the stability of the oxygen evolution complex when inactivated, as it interrupts the flow of electrons in the photosystems [24,31]. This, in turn, explains the greater energy dissipation in the form of heat in the light (*D*) and the lower energy not dissipated or used in the photochemical phase in the light (*Ex*), found in the present study.

Undissipated energy (*Ex*) in Ca^2+^-free plants with mechanical damage may have been responsible for the production of H_2_O_2_ in the chloroplast, as it has been reported by Choudhury et al. [39] and may explain the DAB staining of cells of the palisade parenchyma of these plants. For them, the lower energy produced in the photochemical phase, with less production of reducing agents, may explain the low assimilation rate (*A*), the low rubisco carboxylation efficiency (*A*/*Ci*) and the greater internal carbon accumulation. Previous studies embracing stress revealed the same response when plants were subjected to water deficiency [40,41]. Reduced stomatal conductance (*Gs*), transpiration (*E*) and water use efficiency (iWUE) may be explained by the fact that stomatal opening requires signaling with rapid calcium oscillation, as already addressed by previous studies [42,43], and this movement does not happen in Ca^2+^-free plants, resulting in the stomatal closure.

Thus, it should be noted that growth in the absence of Ca^2+^ promoted an increase in *RBOHD* expression and H_2_O_2_ concentration, being higher when the plants were subjected to mechanical damage, which may have caused oxidative stress contributing to stomatal closure and reduced conductance stomatal (*Gs*). Moreover, the functions of calcium in cell division, cell wall formation, pollen tube to flower fertilization and fruit development [44] explain why plants grown in the absence of Ca^2+^, regardless of mechanical damage, had a low leaf, flower, fruit, stem, and root mass.

Plants grown in the presence of Ca^2+^ and with mechanical damage showed greater expression of *SlCaM2* (Calmodulin gene) six hours after mechanical damage, which suggests that the activation of this gene depends on a lower-level interaction between Ca^2+^ and reactive oxygen species, as revealed in these plants. Thus, calmodulin may be involved with coordinating the action of kinases and antioxidant enzymes in order to make the plant’s response to damage more efficient and rapid, as it has been observed in other studies, which demonstrates that *CaM2* acts in the coordination of kinases and antioxidant enzymes [10,45]. Thus, this may indicate that calcium is essential to control the activity of antioxidant enzymes. The expression of *RBOH1*, *MPK1* and *MPK2* in plants grown with Ca^2+^ and with mechanical damage suggests that the presence of this ion enabled molecular adjustments so that the plants had increased CAT enzyme activity, low levels of hydrogen peroxide and low lipid peroxidation, which can lead to overcoming stress from mechanical damage. Calcium-dependent protein kinases are involved with the *RBOH1* regulation pathway due to its phosphorylation [20,21,22].

Plants grown in the presence of Ca^2+^ showed a reaction to DAB, suggesting that the presence of calcium may have improved peroxidase activity, as indicated by this reaction in tissues [18,46]. In plants without mechanical damage, the main veins and glandular trichomes were marked by DAB, while in those with mechanical damage, the main vein, xylem, palisade parenchyma and glandular trichomes were marked. Staining of trichomes and palisade parenchyma can be explained by high metabolic activity, as presented by Balcke et al. [47], in which case, an increase in peroxidase activity may occur.

As for the photosynthetic process, plants grown in the presence of Ca^2+^ and without mechanical damage showed low minimum fluorescence adapted to the dark (Fo), high capacity to absorb light (Fv’/Fm’), low light fraction absorbed by the PSII antenna dissipated as heat (*D*) and low fraction of excitation energy not dissipated in the antenna and not used for photochemistry in the light (*Ex*), all of which are variables that indicate that the presence of Ca^2+^ allowed, in the light, normal electron flow (ETR) and high quantum productivity (ΦPSII), resulting in the production of reducing agents for carbon reduction [48]. These plants also showed low energy dissipation (*Ex* and *D*), as observed in previous stress studies, due to different causes, among them cadmium [31,37,49,50]. According to the literature, it is suggested that Ca^2+^ triggers the signaling of defense pathways to prevent damage to the photosystem [13]. These conditions contribute to high rates of carbon assimilation, rubisco activity and better control of stomatal opening, with high water use efficiency, as found in other studies [42,43], which is a condition not verified in plants grown in the absence of calcium.

## 4. Materials and Methods

### 4.1. Study Species and Cultivation

*Solanum lycopersicum* “cv. Micro-Tom” seeds were supplied by Lázaro E. P. Peres and germinated in trays with expanded clay of medium texture, according to [51]. Fourteen days after sowing, young plants were grown in standard nutrient solution containing 4 mM Ca^2+^ (control treatment), and Hoagland and Arnon [52] nutrient solution N° 2 modified to provide concentrations of 2 and 0 mM Ca^2+^ (absence of Ca^2+^ or Ca^2+^-free). Seven days later, plants that received mechanical damage had their leaf limb margins cut with the aid of scissors, without removal of plant tissue (Supplementary Figure S2) and kept in the same solutions until later analysis.

The experiment was conducted at the Biostatistics, Plant Biology, Parasitology and Zoology Department of the Biosciences Institute, UNESP, Campus Botucatu, São Paulo, Brazil (22°49′10″ S, 48°24′35″ W, 800 m above sea level) in a paddy fan-greenhouse, with sunrise at 6:20 a.m., temperature maintained at 25.62 ± 2.62 °C and air humidity at 50.35 ± 4.94%. At the moment of all analyses, the photosynthetic photon flux density (PPFD) was at 495.00 ± 75.00 µmol m^−2^ s^−1^ and environmental CO_2_ was at 401.75 ± 23.95 PPM. The experiment used a randomized complete block design with four replications of 16 plants in a 3 × 2 × 5 factorial scheme: 0 (absence of Ca^2+^ or Ca^2+^-free), 2 and 4 mM Ca^2+^, with/without mechanical damage and evaluation times.

### 4.2. Gene Expression

Gene expression of *RBOH1*, *MPK1*, *MPK2* and *SlCaM2* (Table 1) in plants grown in the presence of 0 (absence of Ca^2+^ or Ca^2+^-free) and 2 mM Ca^2+^ were evaluated at 0.5 and 3, 6 and 24 h after mechanical damage to plants, representing the time of greatest gene expression [5,9]. For this, 50 mg of leaves in the region where the mechanical damage was performed were collected at 9:00 a.m., packed in plastic bags, wrapped in aluminum foil and frozen in liquid nitrogen to immediately stop all metabolic reactions. The study of gene expression was performed by Quantitative Polymerase Chain Reaction (* RT-qPCR). The extracted RNA was used to make cDNA by reverse transcription. The presence or absence of a transcript (mRNA) was determined by * RT-qPCR reaction. The extraction of its transcriptome followed the protocol of the manufacturer of the TriZOL Reagent (Thermo Scientific, Waltham, MA, USA). The obtained total RNA samples were quantified and then properly treated with DNase RNase free, according to recommendations (Promega, Madison, WI, USA). The treated RNA samples were used in cDNA synthesis using the High-Capacity kit, according to Thermo Fischer protocol. Subsequently, the cDNA samples were treated with RNase and then used for analysis of *RBOH1*, *MPK1*, *MPK2* and *SlCaM2* by qPCR using the enzymatic system GoTaq^®^ qPCR and RT-qPCR (Table 1). The 2^−ΔΔ*Ct*^ method was used to calculate the level of gene expression (mRNA) of the referred genes. Genes already described in the literature and that had constitutive expression within each treatment were used as normalizers (Table 1). Three biological repetitions for each treatment and three technical repetitions for each biological repetition were performed.

### 4.3. Activity of Antioxidant Enzymes and Quantification of Hydrogen Peroxide and Lipid Peroxidation

Leaves were collected between 9:00 and 11:00 a.m. at 0.5 h and 7, 14 and 21 days after mechanical damage, and samples were frozen in liquid nitrogen and stored until evaluation. Extract was obtained from 100 mg of fresh leaf mass and separated into microtubes and stored at −20 °C for later determination. The activities of the enzymes superoxide dismutase (SOD, EC 1.15.1.1) [55], total peroxidase (POX, EC 1.11.1.7) [56], catalase (CAT, EC: 1.11.1.6) [57] and ascorbate peroxidase (APX, EC: 1.11.1.1) [58] were determined. Total proteins were quantified according to [59], hydrogen peroxide according to Alexieva et al. [60] and lipid peroxidation according to Devi and Prasad [61], with four biological repetitions for each treatment.

### 4.4. Histochemical Analysis of Hydrogen Peroxide

Sections of the median portion of fully expanded leaf blades with mechanical damage at 0.5 h and 21 days after mechanical damage were submitted to histochemical testing with 3,3′-diaminobenzidine (DAB) to locate hydrogen peroxide (H_2_O_2_), following Thordal-Christensen [46], with three biological repetitions for each treatment.

### 4.5. Chlorophyll a Fluorescence, Gas Exchange and Plant Biomass

Chlorophyll a fluorescence was measured between 9:00 a.m. and 11:00 a.m. at 0.5 h and 7, 14, 21 and 28 days after mechanical damage in fully expanded leaves located in the stem region, below mechanical damage, and stored in the dark for 30 min. Measurements were made with a pulse amplitude portable fluorometer (Jr PAM, Walz) under 1150 PPFD saturating irradiance. Minimum dark-adapted fluorescence (Fo), maximum quantum efficiency of photosystem II (Fv/Fm), non-photochemical quenching and estimates of the constant rate of heat loss from PSII [NPQ = (Fm − Fm’)/Fm’] were determined, with four biological repetitions for each treatment.

Chlorophyll *a* fluorescence and gas exchange were evaluated at the same time and in leaves located in the stem region, below mechanical damage, before destructive sampling, using open-system photosynthesis equipment with CO_2_ and water-vapor infrared analyzer and a coupled fluorometer (Infra-Red Gas Analyzer—IRGA, model GFS 3000 FL with LED-Array/PAM-Fluorometer 3055-FL, Walz) at 0.5 h and 7, 14, 21 and 28 days after mechanical damage with saturant light of 1200 µmol m^−2^ s^−1^, 401.75 ± 23.95 PPM and VPD 1.834 ± 0.183 kPa at the moment of analysis. The fluorescence variables measured were effective quantum yield (ΦPSII), electron transport rate (ETR), photosystem II efficiency (Fv’/Fm’), photochemical quenching (qL), light fraction absorbed by the PSII antenna that is dissipated as heat (*D*) and fraction of dissipated excitation energy in the antenna that cannot be used for photochemical phase (*Ex*) [62,63]. The gas exchange variables measured were net CO_2_ assimilation rate (*A*, μmol CO_2_ m^−2^ s^−1^), transpiration rate (*E*, mmol water vapor m^−2^ s^−1^), stomatal conductance (*Gs*, mmol m^−2^ s^−1^) and internal leaf CO_2_ concentration (*Ci*, µmol CO_2_ mol^−1^ ar). Instantaneous water use efficiency (iWUE, μmol CO_2_ (mmol H_2_O^−1^) was determined as the ratio between CO_2_ assimilation rate and transpiration rate (*A/E*), while instant carboxylation efficiency of ribulose enzyme 1,5-diphosphate carboxylase (RuBisCO) was calculated as the ratio between net CO_2_ assimilation rate and internal leaf CO_2_ concentration (*A*/*Ci*) [64], with four biological repetitions for each treatment.

The leaves, stems, roots, flowers, and fruits were subjected to drying at 38 °C until constant dry mass and total dry mass were calculated as the sum of all organs. Leaf area was determined by a leaf area integrator LI-3100C area meter LI-COR at 7, 14, 21 and 28 days after damage, with four biological repetitions for each treatment.

### 4.6. Statistical Analysis

Homogeneity of variances was checked using Levene’s test. The variables were submitted to analysis of variance (three-way ANOVA) and means compared by Tukey’s test at 5% probability (SigmaPlot 12.0) [65]. Heat map generation, hierarchical cluster analysis (HCA) and pairwise correlation heat maps were performed using MetaboAnalyst 4.0 software [66].

## 5. Conclusions

Based on the results presented here and on other studies reported in the literature, damage may be important in activating stress response mechanisms in Ca^2+^-free plants. The signaling of mechanical damage must have been performed by reactive oxygen species, as high levels of H_2_O_2_ and expression of genes involved in stress control and signaling were observed in these plants, which revealed low photosynthetic performance.

Regarding gas exchange, plants grown in the presence of Ca^2+^ and with mechanical damage showed a decrease in the efficiency of instantaneous carboxylation of the activity of the enzyme ribulose 1,5-diphosphate carboxylase (RuBisCO). This result suggests that mechanical damage increased H_2_O_2_, thus generating a signal to activate defensive pathways to reverse stress. Although they did not show the best results in terms of chlorophyll *a* fluorescence and gas exchange, plants cultivated in the presence of Ca^2+^ and subjected to mechanical damage recovered since mechanical damage did not lead to a decrease in total dry mass, which is in agreement with other studies.

We conclude that the plants grown in the absence of a calcium supply recognized the mechanical damage but did not survive. The highest expression of the *RBOH1* gene and the accumulation of H_2_O_2_ in these plants signaled cell death. Plants grown in the presence of calcium showed higher expression of *SlCaM2* and control of H_2_O_2_ concentration, overcoming the stress caused by mechanical damage, with photosynthetic acclimatization and without damage to dry mass production.

## Figures and Tables

**Table 1:** Primers Used in Real-Time Quantification (qRT-PCR).

Gene	Oligonucleotides	Gene ID	Reference
*RBOH1 F*	5′-GGAGCTCCAGCACAAGATTA-3′	Sl08g081690	[18]
*RBOH1 R*	5′-CTTGTTGCAGCACTCATGTC-3′		
*MPK1 F*	5′-GCTGACAGATTGTTGCAGGT-3′	Sl12g019460	
*MPK1 R*	5′-TCCACCCCATAAAGATACATCA-3′		
*MPK2 F*	5′-TACTCGCTCGTTTGCTGTTG-3′	Sl08g014420	
*MPK2 R*	5′-TTGGAGTACAGGAAAACAATGG-3′		
*SlCaM2-F*	5′-TCTGAGGAGGAGTTGAAAGAGG-3′	Solyc10g081170	[9]
*SlCaM2-R*	5′-TCAACATCAGCTTCCCTAATCA-3′		
*Actin F*	5′-GGGATGATATGGAGAAGATATGG-3′	Actina-185 pb	[53]
*Actin R*	5′-AAGCACAGCCTGGATAGC-3′		
*β-6-tubulin F*	5′-TTGGTTTTGCACCACTGACTTC-3′	Tub6-65 pb	[54]
*β-6-tubulin*	5′-AAGCTCTGGCACTGTCAAAGC-3		

## Data Availability

Not applicable.

## Supplementary Materials

The following supporting information can be downloaded at: https://www.mdpi.com/article/10.3390/ijms232113571/s1.
