# Supplementary figures and images for: Roles of Calcium Signaling in Gene Expression and Photosynthetic Acclimatization of Solanum lycopersicum Micro-Tom (MT) after Mechanical Damage
Source: Int J Mol Sci. 2022 Nov 5;23(21):13571. doi: 10.3390/ijms232113571 (PMC9655782; doi:10.3390/ijms232113571)

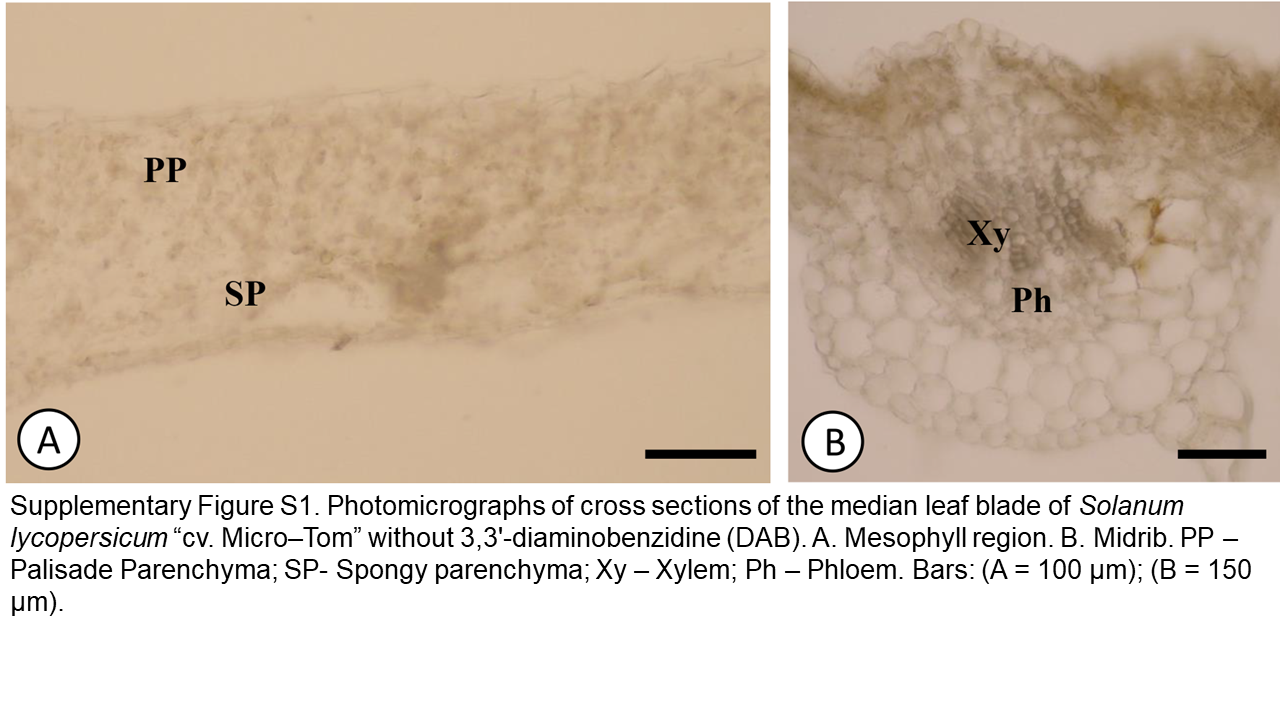

Supplement: Supplementary file 1 [file ijms-23-13571-s001.zip › Supplementary Figure S1_Campos et al.tif]

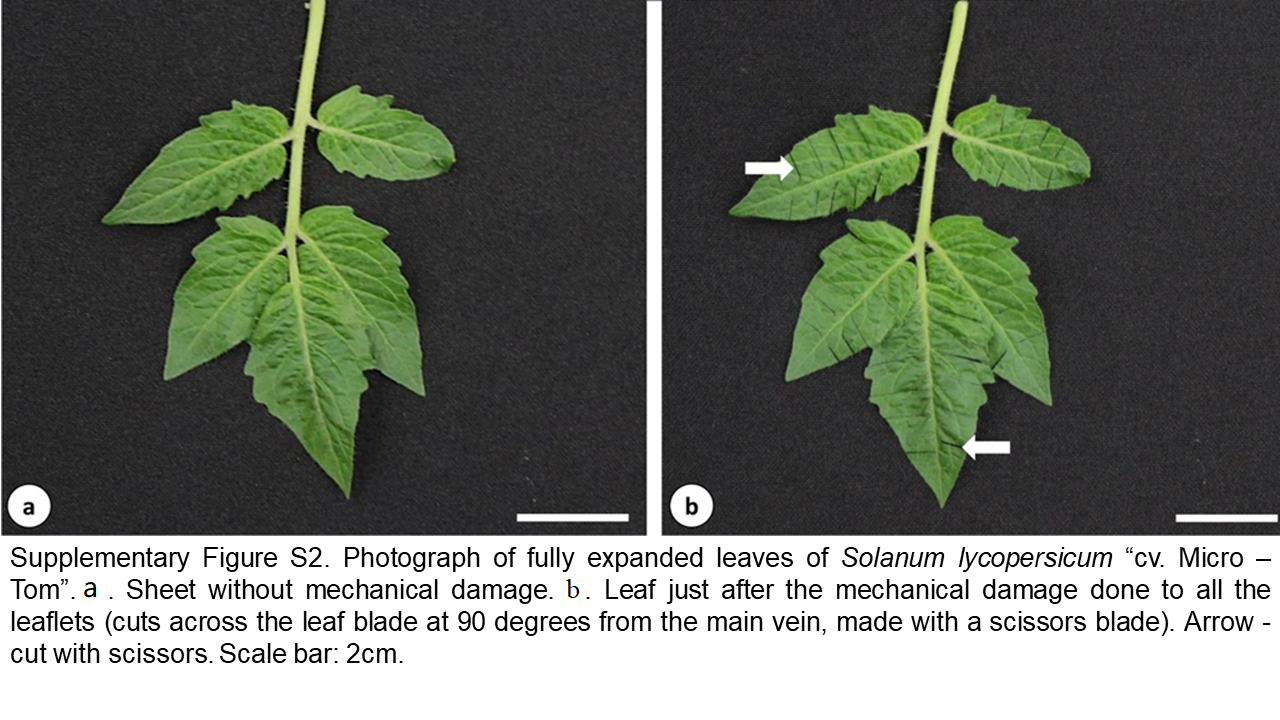

Supplement: Supplementary file 1 [file ijms-23-13571-s001.zip › Supplementary Figures S2_ Campos et al.tif]
